# Supplementary material for: Impact of Small Molecules on Intermolecular G-Quadruplex Formation
Source: Molecules. 2019 Apr 20;24(8):1570. doi: 10.3390/molecules24081570 (PMC6514588; doi:10.3390/molecules24081570)
Supplement: Supplementary file 1 [file molecules-24-01570-s001.pdf]

Supplementary Materials

Impact of Small Molecules on Intermolecular G-quadruplex Formation

Prabesh Gyawali[1], Keshav GC[2], Yue Ma[3], Sanjaya Abeysirigunawardena[2], Kazuo Nagasawa[3], Hamza Balci[1,*],

[1] Department of Physics, Kent State University, Kent, OH, 44242, USA
[2] Department of Chemistry and Biochemistry, Kent State University, Kent, OH, 44242, USA
[3] Department of Biotechnology and Life Science, Tokyo University of Agriculture and Technology, Koganei, Tokyo 184-8588, Japan
* Correspondence: hbalci@kent.edu; Tel.: +1 330 672 2577

Radiolabeling of DNA samples

The 5′-end of the DNA oligonucleotides (S1, S2, S3, and hGQ) were radiolabeled with Adenosine 5′-triphosphate [γ (gamma) - ^{32}P-ATP] (PerkinElmer). DNA oligonucleotides (10 pmoles) was incubated with γ - ^{32}P-ATP (10 µCi) and T4 PNK enzyme (10 units, New England BioLabs) in 1X PNK buffer (provided with the enzyme) at 37 °C for 30 minutes. The reaction was heat inactivated (65 °C for 20 minutes) and passed through a micro spin size exclusion column (Chroma Spin™ TE-10 column, Takara). For DNA oligonucleotide S1, free nucleotides were removed by passing the reaction mixture through a PD MidiTrap™ G-10 gravity column (GE Healthcare). The sequences of the strands used in these assays are as follows:

S1: AGGGT

S2: GGGTTAGGG

S3: AGGGTTAGGGTTAGGGTTAG

hGQ: TTGGGTTAGGGTTAGGGTTAGGGTT

Electrophoretic mobility gel shift assay (EMSA)

In electromobility gel shift assays, increasing concentrations (0-20 µM) of the unlabeled DNA oligonucleotide was added to radiolabeled DNA oligonucleotide (10 nM) in 10 µL of reaction buffer (10 mM Tris-HCl pH 7.5, 150 mM KCl, 2 mM $MgCl_2$). The same reactions were carried out in the presence of 1 µM L1H1-6OTD. iGQ structures were formed by slow cooling for 1 hour at room temperature followed by an initial denaturation step at 90 °C for 5 min. The reaction mixture was mixed with 2 µL of native gel loading buffer and loaded on to a 10% acrylamide gel with 1X TBE-KM buffer (Standard TBE buffer with 150 mM KCl and 2 mM $MgCl_2$). Gels were run for 2 hours at 20 W per gel in a vertical gel electrophoresis set-up equipped with a circulating water bath (-2 °C). After electrophoresis, gels were exposed to the intensifying radioactive screen

overnight. An autoradiograph was recorded using phosphoimaging Typhoon FLA 9500 (GE Healthcare), and the gel image was processed in the ImageJ 1.48v; Java 1.6.0_20 [64-bit] software.

Conditions for the formation of the iGQ complex was optimized by testing several annealing conditions (slow cooling to ambient temperatures followed by incubation at 50, 60, and 90 °C temperatures). The optimal condition was found to be heating at 90 °C for 5 minutes followed by slow cooling to ambient temperatures. Native PAGE gels with several percentages of acrylamide (16% and 10%) were used to control the mobility of the complexes and unbound oligonucleotide sequences. To control the dissociation during the electrophoresis, various running buffer conditions were used. TBE (1mM Tris base, 1mM Boric acid and 0.02 mM Ethylenediaminetetraacetic acid; EDTA), THEM3 (340 mM Tris-base, 660 mM HEPES, 1 mM EDTA and 3 mM $MgCl_2$), and TBE-KM (Standard TBE buffer with 150 mM KCl and 2 mM $MgCl_2$) were used. Interestingly, only TBE-KM buffer system showed a clear shift in mobility. In addition, the labeling scheme was changed (e.g., radiolabel on S3 oligonucleotide vs. S1 oligonucleotide) to observe a clear shift.

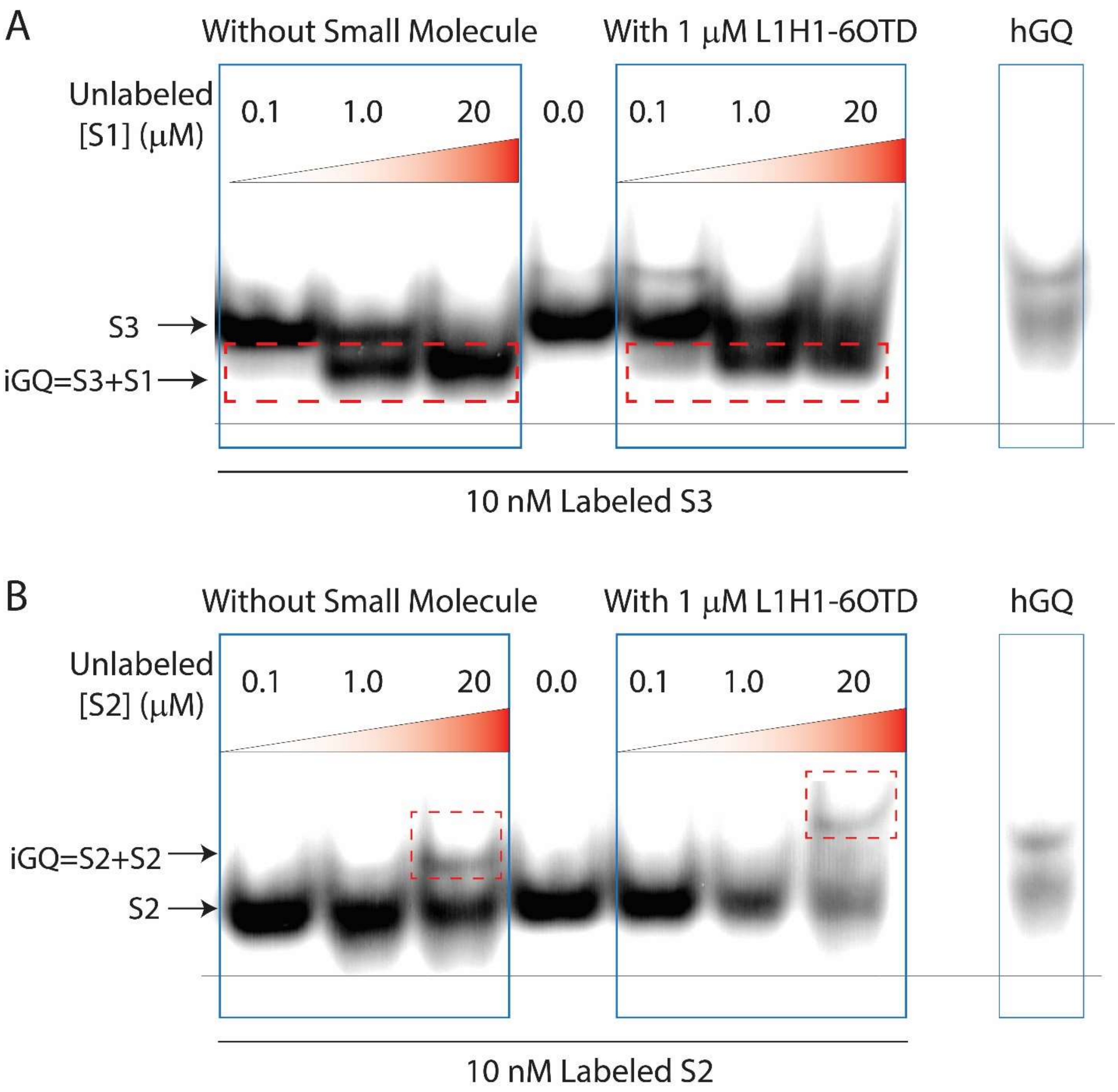

Figure S1: EMSA Measurements using P32 radiolabeled strands. (A) EMSA measurements where P32 radiolabeled S3 strand was kept at 10 nM concentration while S1 was titrated between 1-20 μM concentration in the absence (left panel) and presence of 1 μM L2H2-6OTD. The band representing i-GQ formed between S3 and S1 is indicated with a box with red dashed lines. Due to compaction upon i-GQ formation, the new band runs faster than the S3 band. Intramolecular GQ formed by human telomeric repeat is shown at the far left for reference. (B) Similar EMSA measurements where the concentration of P32 radiolabeled S2 was kept at 10 nM while unlabeled S2 was titrated between 0-20 μM concentration. The band representing i-GQ formed between two S2 strands is indicated with a box with red dashed lines. Intramolecular GQ formed by human telomeric repeat is shown at the far left for reference.
